# Investigating the relationships of different social media platform use with life satisfaction mediated by loneliness: a cross-sectional study

**DOI:** 10.1080/00049530.2026.2753864

**Published:** 2026-10-29

**Authors:** Madeleine Kent, Yu Takizawa

**Affiliations:** a College of Human Sciences and Culture, Flinders University, Adelaide, Australia; b Institute for Mental Health and Wellbeing, Flinders University, Adelaide, Australia

**Keywords:** Social media, social network, loneliness, psychological wellbeing, life satisfaction, young adults

## Abstract

**Objective:**

Existing research rarely distinguishes between different social media platforms, with little investigation into how different platforms relate to psychological wellbeing or how loneliness mediates these relationships. This study investigated the direct and indirect relationships between open network (ONSM) and closed network social media (CNSM) and life satisfaction among young adults with loneliness as a mediator.

**Method:**

Survey data from 1,134 Japanese young adults aged 18–29, including the measures of life satisfaction, loneliness and frequencies of ONSM and CNSM use, were analysed via path-analysis using structural equation modelling to assess direct and indirect relationships between each social media type and life satisfaction, with loneliness as a mediator and demographic variables controlled.

**Results:**

ONSM demonstrated a small but significant negative direct relationship with life satisfaction and a small negative indirect relationship mediated by higher loneliness. In contrast, CNSM showed no direct relationship with life satisfaction but a small positive indirect relationship mediated by lower loneliness.

**Conclusions:**

The present study provides preliminary evidence that lower ONSM engagement and higher CNSM engagement may be related to higher psychological wellbeing, with loneliness identified as a mediator. These insights have practical relevance for promoting healthy social media use. Longitudinal research is needed to establish causation.

## KEY POINTS

What is already known about this topic:

(1) Social media use has inconsistent relationships with psychological wellbeing, with prior research suggesting both positive and negative outcomes depending on patterns and frequency of use.

(2) Loneliness has been identified as a key mediating mechanism in the relationship between social media use and psychological wellbeing, with greater social media use linked to increased loneliness and reduced life satisfaction.

(3) Open and closed network social media platforms differ in their design and social functions, with open networks facilitating broad, diverse connections and closed networks fostering intimate, supportive relationships.

What this topic adds:

(1) This study provides the first direct empirical comparison of open network social media and closed network social media within a single mediation model, demonstrating that their relationships with life satisfaction operate through opposing indirect relationships, with loneliness identified as a mediator.

(2) Findings reveal that open network social media use is directly and indirectly associated with lower life satisfaction, while closed network social media use is exclusively indirectly associated with higher life satisfaction, with loneliness identified as a mediator of this relationship. These cross-sectional findings are consistent with the possibility that the type of online connection is relevant to life satisfaction.

(3) Conducted among Japanese young adults, this study extends predominantly Western literature by providing culturally specific insights into how platform type shapes the relationship between social media use, loneliness, and life satisfaction.

## Introduction

Social media (SM) represent a pivotal innovation in communication technology that has transformed how people interact, access information, and engage with society. SM is an internet-based platform that enables interaction and the distribution of content with both widespread communities and personal networks (Carr & Hayes, 2015). SM serves a variety of purposes, including socialising, information seeking, entertainment, self-expression, business, education, and advocacy (Whiting & Williams, 2013). Beyond individual use, SM extend its influence to shaping social relationships, cultural norms, and even economic activity (Khan, 2025).

As of 2025, the number of active SM users reached approximately 5.24 billion, accounting for 63.9% of the global population (Kemp, 2025). This reflects how deeply embedded SM has become in everyday life, especially among younger generations who represent its primary users (Winstone et al., 2021). The widespread use of SM can be understood through the lens of human motivation. Maslow (1943) identified belonging and esteem as fundamental psychological needs. Accordingly, SM may serve as a contemporary avenue through which individuals seek to fulfil these needs via connection, validation and self-expression. Given the prevalence of SM use, the relationship between SM and psychological wellbeing has attracted considerable public and academic attention.

### Relationship between social media and psychological wellbeing

Research into the relationship between SM and psychological wellbeing has increased over recent decades (Valkenburg, 2022). However, findings remain inconsistent with multiple meta-analyses concluding that while SM use may have a small negative effect, these effect sizes are so small that researchers conclude there is no significant impact (C. Huang, 2017; Kross et al., 2021). Studies included in Marttila et al. (2021) meta-analysis revealed both positive and negative impacts of SM use on psychological wellbeing. Specifically, positive effects include elevated affect, life satisfaction, increased perceived social support, and feelings of connectedness, whereas negative impacts comprise increased negative emotion, higher levels of loneliness, anxiety, and depressive symptoms (Marttila et al., 2021). Such inconsistencies may imply that psychological wellbeing outcomes should not be understood in isolation, yet with consideration to each different platform, pattern, and users’ personal differences.

Kross et al. (2021) suggest that inconsistent findings regarding SM and psychological wellbeing may, in part, be attributed to the diversification of SM platforms. S. Huang et al. (2022) have broadly categorised SM networks into open-network social media (ONSM) and closed-network social media (CNSM). ONSM is primarily designed to allow for interaction and content sharing across broad audiences, including non-followers. Examples of ONSM are Facebook, Instagram, X and LinkedIn. These platforms connect people who may not personally know each other, enabling communication and information exchange with larger communities (S. Huang et al., 2022). Empirical studies indicate ONSM may promote negative outcomes through upward social comparison. For instance, Chou and Edge (2012) found that Facebook users tend to report others’ lives as happier or more successful than their own. Similarly, Verduyn et al. (2015) found that exposure to passive idealised content can result in increased negative self-evaluations. From a theoretical perspective, upward social comparison may intensify these negative outcomes, as exposure to idealised versions of others’ lives can challenge a sense of belonging and psychological wellbeing (Vogel et al., 2014).

In contrast, CNSM refers to platforms primarily designed for users to communicate and share content with known individuals, such as family, friends, and colleagues (S. Huang et al., 2022). Examples include platforms such as WhatsApp, Line, or WeChat. Such platforms are typically private and foster strong ties, reinforcing bonding social capital. Aligned with Maslow’s (1943) premise of belonging, CNSM may satisfy fundamental needs to belong by reinforcing supportive relationships with known connections. These sites prioritise maintaining relationships and privacy between users (S. Huang et al., 2022; Lee & Jung, 2014). Comparatively, ONSM may be less effective in fulfilling these needs.

Distinguishing between these platforms reflects a broad concept of social capital. Social capital refers to resources and benefits that individuals gain from their social relationships, including emotional support, information and opportunities (Ellison et al., 2007). Bridging social capital occurs when connections extend to broader, more diverse networks (Burke et al., 2011), whereas bonding social capital arises from close, supportive relationships that foster trust and a sense of belonging. These ties also satisfy the fundamental human need for connection, reflecting the Need to Belong Theory (Ellison et al., 2007). ONSM platforms facilitate bridging social capital by enabling connections among individuals without preexisting relationships, thereby broadening users’ social networks (Lee & Jung, 2014).

Comparatively, CNSM platforms cultivate bonding social capital, reinforcing intimate and supportive interactions (Ellison et al., 2007). Due to widespread opportunities for varied content consumption, also linked to adverse psychological outcomes, researchers contend that open networks may intensify social comparison (Vogel et al., 2014). Importantly, engagement with open-network platforms may carry greater risks to psychological wellbeing, given increased exposure to comparison and limited emotional closeness with others. Comparatively, closed-network platforms may enhance psychological wellbeing by fostering supportive bonds, grounded in privacy and protection from opportunities for social comparison (Burke et al., 2011). Despite these distinctions, empirical research examining the differential relationships of ONSM and CNSM with psychological wellbeing is limited. Moreover, it remains critical to consider the role that loneliness holds in the relationship between different SM platforms and psychological wellbeing, particularly given consistent findings positioning it as a key mediator of this relationship (S. Huang et al., 2022).

### Loneliness as a mediator of relationship between social media and psychological wellbeing

Loneliness is a subjective negative emotional state that develops from a diminished sense of desired companionship or interpersonal relationships (Mao et al., 2023). Through the lens of the Need to Belong Theory, loneliness represents the aversive feeling when the fundamental human need for close, meaningful relationships is unmet (Baumeister & Leary, 2017). This theoretical perspective highlights why loneliness may be a key psychological mechanism in the link between SM and psychological wellbeing.

Given that prior research indicates that SM influences psychological wellbeing, citing loneliness as a mediator, it remains important to better understand such findings. For instance, Lin et al. (2022) found that social network site use is negatively associated with loneliness, where higher use leads to lower loneliness, which in turn, predicted lower life satisfaction. Similarly, Marttila et al. (2021) reported that problematic (compulsive and uncontrollable) SM use increased loneliness and, in turn, reduced life satisfaction. This highlights an indirect relationship through loneliness that influences the decrease in life satisfaction; however, not on its own. Similarly, Mao et al. (2023) assessed the interaction between SM use and loneliness. Findings revealed that SM use can increase and decrease loneliness in participants, depending on whether interpersonal needs are satisfied through interactions.

Given that ONSM primarily facilitates bridging social capital through broad and often superficial communication, it may expose users to upward social comparison and undermine a sense of belonging (S. Huang et al., 2022). Consequently, greater ONSM use may be associated with increased loneliness and, in turn, reduced psychological wellbeing. In contrast, CNSM primarily cultivates bonding social capital by reinforcing intimate and supportive interactions (Ellison et al., 2007), and greater CNSM use may therefore be associated with decreased loneliness and higher psychological wellbeing. Despite these converging theoretical and empirical grounds, no research has directly examined whether loneliness differentially mediates the relationships between ONSM and CNSM use and psychological wellbeing.

### Theoretical framework

This study draws primarily on the Need to Belong Theory (Baumeister & Leary, 2017), which holds that humans have a fundamental motivation to form and maintain frequent, positive, and stable interpersonal relationships. When this need goes unmet, the result is loneliness: an aversive emotional state with well-documented consequences for psychological wellbeing (Baumeister & Leary, 2017). Loneliness is therefore treated in this study as a psychological mechanism, not simply an outcome, linking social media use to life satisfaction. ONSM and CNSM are expected to differ in how well they meet this need. CNSM fosters bonding social capital through close, reciprocal relationships and may more directly satisfy belonging needs, while ONSM fosters bridging social capital through broader, shallower connections and may be less effective in doing so (Ellison et al., 2007). These expectations underpin the hypotheses that follow.

### Current study

Addressing calls from Kross et al. (2021) and S. Huang et al. (2022) to examine social media’s differential effects by platform type, the present study aims to evaluate the differential relationship of ONSM and CNSM on psychological wellbeing, both directly and indirectly through the mediating role of loneliness among young adults aged 18–29. Life satisfaction is one of the key indicators of psychological wellbeing, defined as an individual’s cognitive evaluation of their overall life circumstances, based on personal standards of happiness, contentment, and fulfilment (Diener, 1984). This study focuses on life satisfaction as the primary outcome variable, given its widespread use as a key indicator of psychological wellbeing across prior literature (Diener et al., 2018; Vittersø, 2025). While SM use is becoming more prevalent across different age groups, young adults aged 18–29 remain the primary and most engaged users of SM (Kemp, 2025). Accordingly, the present study focused on investigating the relationship of ONSM and CNSM within this population. Conducting this study provides novel insights into how different types of SM relate to life satisfaction and the mediating role of loneliness. Understanding these mechanisms may inform interventions and educational programs to promote healthy SM use.

To examine the direct and indirect relationships between ONSM and CNSM use and life satisfaction, with loneliness as a mediator, the following hypotheses were developed:

H1. Greater frequency of engagement with the ONSM platforms will be negatively associated with life satisfaction, indicating a direct relationship among young adults.

H2. Greater frequency of engagement with CNSM platforms will be positively associated with life satisfaction, indicating a direct relationship among young adults.

H3. Greater frequency of engagement with ONSM platforms will be indirectly associated with lower life satisfaction, with loneliness tested as a mediator among young adults.

H4. Greater frequency of engagement with CNSM will be indirectly associated with higher life satisfaction, via loneliness tested as a mediator among young adults.

## Materials and methods

### Ethical approval

Ethical approval was not required for this study, as archival open-access data were utilised.

### Study design

This study employed a cross-sectional research design using quantitative methods to examine the direct relationship between ONSM use, CNSM use, and life satisfaction, as well as the indirect relationships between ONSM use, CNSM use and life satisfaction mediated by loneliness.

### Participants

Participants consisted of 1,134 Japanese young adults (553 men, 576 women, and 5 who indicated “prefer not to answer”) aged 18 to 29 years (see Table 1). They were recruited for an online survey by the Japanese market research company My Voice Corporation (https://www.myvoice.co.jp/). Participation was voluntary, and participants received a small monetary incentive of JPY200 (AUD $2.10) for their time. To ensure an accurate representation of the Japanese population, data were collected from adults residing in two major regions of Japan: Kanto and Kinki.

**Table 1.** Sample demographics.

Variables	*n*	%
Gender		
Male	553	48.8
Female	576	50.8
No response	5	0.4
Age		
18–21	283	24.96
22–25	296	26.11
26–29	555	48.95
Geographic location Kanto region prefectures		
Tokyo	321	28.3
Kanagawa	179	15.8
Saitama	135	11.9
Chiba Kinki region prefectures	112	9.9
Osaka	163	14.4
Kyoto	46	4.1
Nara	27	2.4
Wakayama	14	1.2
Shiga Hyogo	19 118	1.7 10.4
Marriage status		
Married	166	14.6
Not married	968	85.4
Employment status		
Employed/students	996	87.8
Unemployed	138	12.2
Personal income		
No income	174	15.3
>JPY1,200,000	374	33.0
JPY1,200,000– JPY2,400,000	101	8.9
JPY2,400,000-JPY3,600,000	172	15.2
JPY3,600,000-JPY4,800,000	139	12.3
JPY4,800,000- JPY6,000,000	85	7.5
JPY6,000,000-JPY7,200,000	32	2.8
JPY7,200,000-JPY8,400,000	7	0.6
JPY8,400,000-JPY12,000,000	5	0.4
JPY12,000,000<	4	0.4
No response	41	3.6

### Procedure

My Voice Corporation (https://www.myvoice.co.jp/), a market research company, conducted an online survey for adults who are registered as monitors of the company. Participants were recruited through an open-source online survey collected on behalf of the Yoshida Hideo Memorial Foundation (https://www.yhmf.jp/en/). Data were collected in April 2024 and have since been made publicly available as part of ongoing market research on the webpage of the Yoshida Hideo Memorial Foundation. Five thousand and eighty participants, aged 18–64 years, were recruited for this survey. Participants completed an online survey lasting 30 minutes, answering questions concerning SM use, loneliness, and life satisfaction. To maintain a focus on a young adult population, researchers exclusively extracted and used the data of 1,134 participants, aged 18–29 years, downloaded from the webpage in compliance with the guidelines of the use of archival data published by the Yoshida Hideo Memorial Foundation.

### Measures

#### Overview

The online survey measured life satisfaction, loneliness, and frequency of use of ONSM and CNSM among participants. The online survey also inquired about demographic information, including age, gender, personal income, geographic location, employment status, and marital status.

#### Life satisfaction

The Satisfaction with Life Scale (SWLS) was used to measure subjective life satisfaction (Diener et al., 1985). The SWLS is one of the most widely used measures of life satisfaction across international research, suitable for individuals aged 10 and above (Pavot & Diener, 2008). This five-item self-report scale assesses an individual’s judgment of life satisfaction. Items are rated on a five-point Likert scale (1 = strongly disagree, 5 = strongly agree). Example items include *“In most ways my life is close to my ideal”* and *“I am satisfied with my life”*. The total score ranges from 5 to 25, where higher scores indicate greater life satisfaction. A Japanese version of the SWLS developed by Sumino (1994) was utilised in this study. This measure demonstrates excellent internal reliability (*α* = .90) and convergent validity, showing significant correlations with self-esteem measured by the Rosenberg self-esteem measure *r* = .59, (Rosenberg, 1965) and with subjective happiness measured by the Subjective Happiness Scale *r* = .63, (Yoshimori, 1992).

#### Loneliness

The University of California, Los Angeles (UCLA) Loneliness Scale version 3 (UCLA-LS3) was used to measure loneliness (Russell, 1996). This is a 20-item self-report measure that assesses subjective feelings relating to loneliness (Russell, 1996). This measure comprised 10 negatively framed questions and 10 positively framed questions. Questions on this scale use a four-point Likert scale (1 = never, 4 = often). Example items include *“I feel isolated from others”* and *“There are people I can talk to”*. The total score ranges from 20 to 80, where greater scores indicating greater loneliness. The UCLA-LS3 was employed, as it is widely used across international research to measure loneliness in adults (Russell, 1996). For this study, the Japanese version of the UCLA-LS3 was administered (Masuda et al., 2012). In support of this measure, a validation study among a Japanese adult population reported excellent internal reliability (*α* = .92) and divergent validity (*r* = −.58 with quality of social network) (Masuda et al., 2012).

#### Use of open and closed-network social media

The two SM types of focus are ONSM and CNSM. Participants reported their use according to the type of SM they engage with, regardless of the specific platform. Additionally, this approach allowed a broader understanding of SM use, rather than limiting data relating to a single SM site. To measure the frequency of use for ONSM and CNSM, participants were asked two original questions. The questions used a five-point Likert scale (1 = do not use at all, 2 = hardly use it, 3 = sometimes use it, 4 = use it frequently, 5 = use it very frequently) and comprised the following: Q1: *How often do you use open-network social media (Facebook, X, and/or Instagram)?* Q2: *How often do you use closed-network social media (LINE, social network limited to family, friends, and/or acquaintances)?*

### Data-analysis

The descriptive statistics were calculated for the study variables, including ONSM and CNSM use, life satisfaction and loneliness, as well as for control variables, including age, gender, personal income, geographical location, employment status, and marital status.

To test H1-4, a path analysis was conducted using structural equation modelling (SEM) to assess both the direct and indirect relationships of ONSM and CNSM use with life satisfaction, with loneliness serving as a mediator (see Figure 1). Control variables were incorporated into the model to account for potential confounding factors. This allowed for a more rigorous evaluation of the unique contributions of each type of SM use to life satisfaction, both directly and through the mediating role of loneliness. Standardised beta coefficients were interpreted according to Cohen’s guidelines: small (*β* = .10), medium (*β* = .30), and large (*β >*.50). A significance level of .05 was applied throughout all analyses. All statistical analyses were conducted using IBM SPSS Statistics version 30 (IBM Corp., 2024) and Mplus version 8.11 (Muthén & Muthén, 2017, 2023). As the design is cross-sectional, loneliness was tested as a mediator of the association between SM use and life satisfaction. This approach identifies the extent to which loneliness accounts for the association but does not establish the temporal or causal sequence among variables.

**Figure 1.**
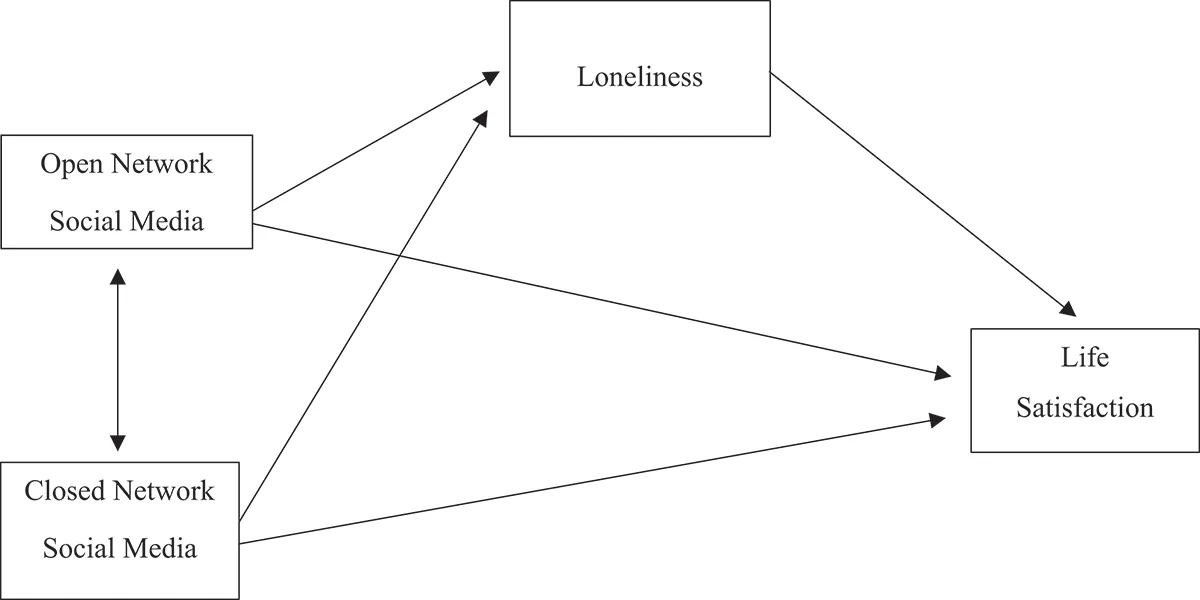
Hypothesised mediation model.

Following guidelines for SEM, Wolf et al. (2013) recommend a sample size of 200 to 500 for models with fewer than 30 parameters, and Jackson (2003) suggests an n:q ratio of 20:1, yielding a minimum recommended size of 200. The present sample (*n* = 1,134) substantially exceeds this threshold, providing sufficient statistical power and stability to estimate model parameters, reduce standard error, and minimise risks of convergence issues and biased estimates (Cohen, 1988).

## Results

### Descriptive statistics

The online survey was completed by 1,134 participants. Participants were required to complete all survey items except for the demographic variables; all survey items relating to predictor and outcome variables had a 100% response rate. Data were missing from five participants (0.44%) in the gender survey question. Data within the personal income survey question were missing from 41 participants (3.62%).

Table 2 presents the descriptive statistics for the study variables. Cronbach’s alpha for the life satisfaction and loneliness measures ranged from .90 to .92, respectively. This indicates excellent internal reliability for both scales. Participants reported moderate use of ONSM and low to moderate use of CNSM. The mean life satisfaction scores amongst participants were moderate (*M* = 14.44, SD = 4.60). Similarly, the mean score for loneliness amongst participants was slightly elevated (*M* = 51.25, SD = 10.05). The descriptive statistics revealed that several control variables exhibited skewed distributions. Marital status (skewness = 2.00; kurtosis = 2.02) showed a substantial, significant positive skew, and employment status (skewness = −2.32; kurtosis = 3.38) showed a substantial, significant negative skew. These deviations suggest potential violations of the assumption of normality. To mitigate this, bootstrapping with 1,000 resamples was employed during the structural equation modelling (SEM) analyses, as is recommended for addressing non-normal data (Ory & Mokhtarian, 2010). Skewness values for all other variables ranged from −2 to +2, suggesting an acceptable approximation to normal distributions. Pearson’s correlations for all study variables are displayed in Table 3.

**Table 2.** Descriptive results for study variables.

Variables	*M*	SD	Range	Skewness	Kurtosis	*α*
Life satisfaction	14.44	4.60	5–25	−.27	−0.14	.90
Loneliness	51.25	10.05	20–80	.18	1.36	.92
ONSM use	3.41	1.50	1–5	−0.50	−1.18	
CNSM use	2.89	1.45	1–5	−0.01	−1.34	
Age	24.61	3.58	18–29	−0.47	−1.10	
Gender (Female)	0.51	0.50	0–1	−0.04	−2.00	
Geographical location (Kanto)	0.34	0.47	0–1	0.67	−1.55	
Marital status (Married)	0.15	0.35	0–1	2.00	2.02	
Employment status (Employed)	0.88	0.33	0–1	−2.32	3.38	
Personal income	2.19	1.81	0–9	0.80	0.10	

Note: ONSM use = open network social media use, CNSM use = closed network social media use.

**Table 3.** Correlation between open network and closed network social media use, loneliness, life satisfaction and control variables.

	LO	LS	ONSM	CNSM	Gender	Age	Marital Status	Personal Income	Employment Status	Prefecture	Region
LO											
LS	−.45**										
ONSM	.04	−.09**									
CNSM	−.10**	.02	.51**								
Gender	.01	−.03	.06	.05	–						
Age	−.02	−.06*	−.06*	−.11**	−.05	–					
Marital Status	−.14**	.13**	−.05	−.05	.12**	.32**	–				
Personal income	−.15**	.19**	−.01	.06	−.16**	.48**	.19**				
Employment status	−.19**	.21**	−.01	.11**	.07*	−.05	.14**	.32**			
Prefecture	−.04	.01	−.01	.02	.02	.00	.09**	−.05	−.01	–	
Region	−.04	.01	−.01	.02	.03	.00	.10**	−.05	−.02	.99**	

Note: **p < .01. *p < .05. *LS* = Life satisfaction. *LO* = Loneliness. *ONSM* = Open network social media. *CNSM* = Closed network social media.

### Path analysis using structural equation modelling

#### Overview

A path analysis using structural equation modelling was conducted to test the hypothesised relationships. The model was estimated using maximum likelihood (ML) in Mplus. As the analysis only examined the relationship between measured variables without creating latent variables, model fit indices indicated a perfect fit, χ^2^(0) = 0.00, *CFI* = 1.00, *TLI* = 1.00, *RMSEA* = .00, and *SRMR* = .00 (Browne & Cudeck, 1993). According to recommended cut-offs, these values indicate the model accurately captures the patterns of the hypothesised relationships (West et al., 2012). This model explained an overall 25.7% of variance in life satisfaction (*R*^2^ = .26), 7.2% in loneliness (*R*^2^ = .07), 3.4% in CNSM use (*R*^2^ = .03), and 0.9% in ONSM use (*R*^2^ = .01).

### Direct relationships of open and closed-network social media with life satisfaction

The direct relationships of ONSM and CNSM with life satisfaction and loneliness are presented in Table 4. Independent of the influences of demographic variables and frequency of CNSM use, ONSM use had a small, significant negative relationship with life satisfaction (*β* = −.23, *p* = .010) and a large, significant positive relationship with loneliness (*β* = .63, *p* = .005). However, CNSM use had no relationship with life satisfaction (*β* = −.03, *p* = .799). In contrast, CNSM showed a large, significant negative relationship with loneliness (*β* = −.88, *p* < .001). Loneliness was also found to be a small, significant negative predictor of life satisfaction (*β* = −.19, *p* < .001), indicating that higher loneliness is associated with lower life satisfaction. These direct relationships provide partial support for the hypothesised mediation model, in which loneliness may account for part of the relationship between SM use and life satisfaction. The direct relationship of ONSM use with life satisfaction is consistent with Hypothesis 1, suggesting that greater ONSM engagement is associated with lower life satisfaction. However, CNSM use was not directly associated with higher life satisfaction.

**Table 4.** Direct relationships of ONSM and CNSM use with loneliness and life satisfaction.

Outcome		Predictor	*β*	*SE*	*95% CI*
Life satisfaction	←	Loneliness	−.19**	.01	(−0.21, −0.16)
Life satisfaction	←	ONSM	−.23*	.09	(−0.41, −0.06)
Life satisfaction	←	CNSM	−.03	.10	(−0.23, 0.18)
Loneliness	←	ONSM	.63**	.23	(0.19, 1.08)
Loneliness	←	CNSM	−.88**	.25	(−1.37, −0.39)

Note: *ONSM* = Open-network social media, *CNSM* = Close-network social media. *p < .05, **p < .01.

Table 5 presents the relationships of control variables (gender, age, marital status, geographical location, employment status and personal income) with life satisfaction and loneliness, and SM use.

**Table 5.** Relationships of control variables with ONSM, CNSM, loneliness, and life satisfaction.

	Gender (female)			Age		
	*β*	*SE*	95% *CI*	*β*	*SE*	95% *CI*
Life satisfaction	−.19	.24	[−0.66, 0.29]	−.23**	.04	[−0.31, −0.15]
Loneliness	.34	.60	[−0.84, 1.52]	.17	.10	[−0.03, 0.36]
ONSM	.20*	.09	[0.03, 0.38]	−.03	.02	[−0.06, 0.01]
CNSM	.19*	.09	[0.01, 0.37]	−.06**	.02	[−0.09, −0.03]
	Marital status			Geographical location (Kanto)		
	*β*	*SE*	95% *CI*	*β*	*SE*	95% *CI*
Life satisfaction	1.19**	.38	[0.44, 1.95]	−.01	.08	[−0.17, 0.16]
Loneliness	−3.39**	.87	[−5.10, −1.68]	−.24	.20	[−0.63, 0.15]
ONSM	−.19	.14	[−0.47, 0.09]	−.00	.03	[−0.06, 0.06]
CNSM	−.19	.13	[−0.46, 0.07]	.03	.03	[−0.03, 0.09]
	Employment status			Personal income		
	*β*	*SE*	95% *CI*	*β*	*SE*	95% *CI*
Life satisfaction	.75*	.38	[0.01, 1.49]	.43**	.09	[0.26, 0.60]
Loneliness	−3.85**	1.12	[−6.05, −1.64]	−.58**	.19	[−0.96, −0.21]
ONSM	−.10	.16	[−0.41, 0.22]	.03	.03	[−0.04, 0.10]
CNSM	.28*	.14	[0.00, 0.56]	.10**	.03	[0.03, 0.16]

Note: ONSM = Open-network social media, CNSM = Close-network social media. *p < .05, **p < .01.

### Indirect relationships of open and closed-network social media with life satisfaction via loneliness

Table 6 displays the indirect relationships of ONSM and CNSM with life satisfaction, mediated by loneliness. ONSM showed a small, significant, negative indirect relationship with life satisfaction via loneliness (*β* = −.12, *SE* = .04, 95% *CI* [−0.20, −0.03]), indicating that loneliness accounted for part of the association between greater ONSM use and lower life satisfaction. This is consistent with Hypothesis 3. In contrast, CNSM demonstrated a small, significant positive indirect relationship with life satisfaction through loneliness (*β* = 0.16, *SE* = .05, 95% *CI* [0.07, 0.26]), indicating that loneliness accounted for part of the association between greater CNSM use and higher life satisfaction, consistent with Hypothesis 4.

**Table 6.** Indirect relationships of ONSM and CNSM use with loneliness and life satisfaction.

Outcome		Mediator		Predictor	*β*	*SE*	95% *CI*
Life satisfaction	←	Loneliness	←	ONSM	−.12*	.04	[−0.20, −0.03]
Life satisfaction	←	Loneliness	←	CNSM	.16**	.05	[0.07, 0.26]

Note: ONSM = Open-network social media, CNSM = Close-network social media. *p < .05, **p < .01.

## Discussion

This study examined the relationships between ONSM and CNSM use and life satisfaction, with loneliness as a mediator, among Japanese young adults. A novel contribution is the direct comparison of two functionally distinct SM types within a single mediation model. This provides empirical evidence that the relationships between SM use and psychological wellbeing vary meaningfully by platform type.

Before discussing specific relationships, it should be noted that loneliness was tested as a mediator within a cross-sectional design. This approach can identify whether loneliness accounts for part of the association between SM use and life satisfaction. However, it cannot establish the temporal order of these variables or confirm a causal mechanism. ONSM use, CNSM use, loneliness, and life satisfaction were all measured concurrently. The findings are therefore consistent with the forward-pathway interpretation emphasised in our hypotheses, but are equally consistent with loneliness preceding or co-occurring with SM use, or with a reciprocal relationship between the two over time. The discussion that follows interprets the mediating role of loneliness in light of relevant theory, while acknowledging that these alternative directions of influence cannot be ruled out by the present data.

### Open network social media by life satisfaction and the mediating role of loneliness

Consistent with Hypothesis 1, ONSM use demonstrated a small but significant negative direct relationship with life satisfaction. Importantly, this relationship persisted after accounting for loneliness. This extends existing literature by suggesting that mechanisms beyond loneliness contribute to this association (C. Huang, 2017; Vogel et al., 2014). One key explanation draws on Social Comparison Theory (Festinger, 1954). ONSM platforms frequently expose users to curated, idealised portrayals of others’ lives. This fosters upward social comparison, undermines self-esteem, and reduces life satisfaction (Vogel et al., 2014). Other unmeasured mechanisms may also contribute, including SM addiction (Kuss & Griffiths, 2017), quality of interaction (Sun et al., 2020), and exposure to emotionally charged algorithmic content (Gombar, 2025). As none of these mechanisms were directly measured in this study, this direct relationship should be interpreted cautiously pending further investigation.

It is worth noting that not all prior research has found consistent associations between SM use and psychological wellbeing. Several meta-analyses have concluded that effect sizes are too small to indicate a meaningful impact (C. Huang, 2017; Kross et al., 2021). The present findings suggest that platform-differentiated analysis may help explain some of this inconsistency: aggregating across ONSM and CNSM, which show opposing associations with life satisfaction in this study, could obscure meaningful effects when SM use is treated as a single, undifferentiated construct in prior work.

Consistent with Hypothesis 3, greater ONSM use was indirectly associated with lower life satisfaction through increased loneliness. Notably, this indirect pathway was identified within a Japanese young adult sample, a population underrepresented in existing SM and wellbeing research. This adds cultural specificity to a predominantly Western literature (Lin et al., 2022; Mao et al., 2023; Marttila et al., 2021). Baumeister and Leary’s (2017) Need to Belong Theory offers a useful explanatory framework. While ONSM provides broad social engagement, its interactions are theorised to be largely superficial, which may fail to satisfy the need for meaningful connection, consistent with elevated loneliness among more frequent ONSM users.

It should be noted that interaction quality was not directly measured in this study. This explanation draws on theoretical accounts of ONSM engagement rather than on data collected here. Although ONSM can support bridging social capital and weak ties (Burke et al., 2011; Ellison et al., 2007), these connections appear insufficient to foster the deeper bonding necessary to mitigate loneliness. Because the design is cross-sectional, this pattern is equally consistent with an alternative direction of influence: individuals experiencing greater loneliness may be more inclined to seek connection through ONSM in the first place, or loneliness and ONSM use may reinforce one another reciprocally over time (Lin et al., 2022; Marttila et al., 2021). The present data cannot distinguish between these possibilities, and both merit consideration alongside the account offered by Need to Belong Theory.

### Closed network social media by life satisfaction and the mediating role of loneliness

Contrary to Hypothesis 2, CNSM use did not demonstrate a significant direct relationship with life satisfaction. This departs from prior assumptions that platforms supporting close relationships and bonding social capital are directly associated with enhanced wellbeing (Putnam, 2000). Instead, findings are consistent with the possibility that the association between CNSM and life satisfaction operates primarily through reduced loneliness, at least within the mechanisms tested here. This represents an important theoretical refinement: the benefits of close-network SM engagement may be indirect, operating through reduced social isolation rather than through any direct association with wellbeing. Participants also reported relatively modest CNSM use (*M* = 2.89 on a five-point scale), which may have attenuated a direct relationship. Future research should examine whether higher levels of engagement alter this pattern.

Consistent with Hypothesis 4, CNSM use was indirectly associated with higher life satisfaction through reduced loneliness. This stands in direct contrast to the ONSM indirect association, where loneliness mediated a negative association with life satisfaction. This opposing pattern across the two SM types is a key contribution of this study. It demonstrates that ONSM and CNSM are associated with qualitatively different relationships with loneliness and, in turn, with life satisfaction. Unlike ONSM, CNSM’s association with lower loneliness is consistent with its role in facilitating bonding social capital and emotionally supportive ties, which may in turn support life satisfaction (Burke & Kraut, 2016; Ellison et al., 2007; Putnam, 2000). As with the ONSM pathway, however, the reverse pattern, in which less lonely individuals engage more readily with CNSM, cannot be ruled out given the cross-sectional design. Consistent with the Need to Belong Theory (Baumeister & Leary, 2017), CNSM may help satisfy the need for meaningful connection, particularly for those who struggle to maintain close relationships offline. Nevertheless, not all CNSM interactions foster genuine emotional depth. Individual differences in social skills, personality, and offline relationship quality may moderate these associations.

### Cultural considerations

The cultural context of the sample adds an additional layer of novelty to these findings. In collectivist societies such as Japan, social norms around Honne (true self) and Tatemae (public self) shape both online and offline behaviour (Markus & Kitayama, 2014). On ONSM, cultural pressures toward curated self-presentation may amplify upward social comparison, which is consistent with the greater loneliness and lower life satisfaction observed among more frequent users. On CNSM, interactions may be shaped by social obligation rather than authentic emotional exchange. This interpretation is speculative, as the nature and quality of CNSM interactions were not assessed in the present study. This could explain why CNSM was associated with reduced loneliness but not directly with greater life satisfaction. These findings suggest that the mechanisms linking SM use to wellbeing may operate differently across cultural contexts. Cross-cultural replication is needed to determine whether these pathways generalise beyond collectivistic contexts.

### Implications

These findings carry both theoretical and practical significance, though we emphasise that the practical recommendations below are necessarily provisional given the cross-sectional design and should be treated as hypotheses for future longitudinal and intervention research rather than as established guidance. Theoretically, this study demonstrates that ONSM and CNSM are associated with opposing indirect relationships with life satisfaction through loneliness, challenging assumptions of uniform SM associations and highlighting the critical importance of distinguishing between platform types in SM research. Methodologically, this study also demonstrates the value of large-scale archival datasets for testing platform-differentiated mediation models with adequate statistical power, offering a template for future secondary-data research in this area. Practically, these findings offer potentially valuable insights across multiple sectors.

Given the correlational nature of these findings, any practical application should be treated as provisional. Clinicians working with young adults experiencing loneliness could consider discussing platform-specific patterns of social media use as one factor among many worth exploring, rather than as an established intervention target. Educators might embed platform-specific digital literacy into school and university wellbeing programmes to support informed social media choices. Policymakers could draw on these findings as one input among several when considering public health messaging that moves beyond general screen time recommendations. Platform developers may find these patterns relevant when considering design features that support authentic, reciprocal interaction, though this remains speculative pending experimental confirmation.

However, these recommendations warrant caution. Excessive CNSM engagement may foster dependency, social obligation, or compulsive use patterns (Tullett-Prado et al., 2023). This could reduce opportunities for offline relationship development and contribute to stress. Any consideration of CNSM engagement as a wellbeing-relevant factor, therefore, requires careful attention to individual differences, cultural context, and the balance between online and offline social engagement.

### Limitations

The use of a Japanese young adult sample limits generalisability. Cultural factors unique to Japan, such as collectivist values and Japan-specific SM platform preferences, may shape the relationships between SM use, loneliness, and life satisfaction in ways that do not apply to other contexts. Future research should replicate these findings across diverse cultural settings and broader age ranges. The cross-sectional design is a central limitation of this study. While loneliness was tested as a mediator of the association between SM use and life satisfaction, this design cannot establish temporal precedence among ONSM/CNSM use, loneliness, and life satisfaction, nor distinguish the hypothesised forward pathway from reverse causation (e.g., loneliness increasing SM use) or from a reciprocal, bidirectional relationship unfolding over time. Accordingly, the mediating role of loneliness identified in this study should be understood as a finding consistent with, rather than confirmation of, a causal mediating process. Longitudinal and experimental designs, for example, cross-lagged panel models or ecological momentary assessment, are needed to test the directionality assumed by the mediation framework.

Reliance on self-reported SM use may also have introduced recall bias, and objective measures such as digital tracking could offer greater precision in future studies. Additionally, ONSM and CNSM use were each assessed using a single self-report item capturing frequency of use, which precludes estimation of internal reliability and may not fully capture the range of behaviours within each platform category, particularly given that quite different platforms (e.g., Instagram and X) were grouped under a single ONSM item. Given the complexity and multidimensional nature of social media engagement, this approach also provides only a limited representation of the constructs under investigation. Important dimensions such as active versus passive engagement, interaction quality, exposure to social comparison, and platform-specific behaviours were not directly assessed. As such, explanations offered in the Discussion that draw on these dimensions should be understood as theoretically grounded interpretations rather than measured findings. Future studies would benefit from validated multi-item measures that capture these dimensions and distinguish between individual platforms. Finally, exploring additional mechanisms may further explain variance in life satisfaction. Alternative mediators such as fear of missing out and self-esteem, and moderators such as personality, socioeconomic status, and quality of online interaction, warrant investigation to provide a more nuanced understanding of how different forms of SM use influence psychological wellbeing.

## Conclusions

The present study provides novel insights into the complex relationships between social media platform types, loneliness, and psychological wellbeing. Findings suggest that reduced ONSM use and increased CNSM use are each associated with higher life satisfaction, with loneliness identified as a mediator of these associations, underscoring the importance of distinguishing between platform types when examining psychological outcomes. While longitudinal research and replication across broader and more diverse populations are needed to confirm causation and generalisability, these findings could offer useful directions for clinicians, educators, and policymakers seeking to promote healthy social media use and support the psychological wellbeing of young adults.

## Data Availability

All data used in this study are publicly available from the Yoshida Memorial Foundation (https://www.yhmf.jp/en/)
